# Expression Profiling of the Transient Receptor Potential Vanilloid (TRPV) Channels 1, 2, 3 and 4 in Mucosal Epithelium of Human Ulcerative Colitis

**DOI:** 10.3390/cells7060061

**Published:** 2018-06-15

**Authors:** Theodoros Rizopoulos, Helen Papadaki-Petrou, Martha Assimakopoulou

**Affiliations:** Department of Anatomy, Histology and Embryology, School of Medicine, University of Patras, Rion 26504, Greece; thodrizop@gmail.com (T.R.); elenipetroo2000@yahoo.gr (H.P.-P.)

**Keywords:** TRPV1, TRPV2, TRPV3, TRPV4, mucosal epithelium, ulcerative colitis, inflammatory bowel disease

## Abstract

The Transient Receptor Potential (TRP) family of selective and non-selective ion channels is well represented throughout the mammalian gastrointestinal track. Several members of the Transient Receptor Potential Vanilloid (TRPV) subfamily have been identified in contributing to modulation of mobility, secretion and sensitivity of the human intestine. Previous studies have focused on the detection of TRPV mRNA levels in colon tissue of patients with inflammatory bowel disease (IBD) whereas little information exists regarding TRPV channel expression in the colonic epithelium. The aim of this study was to evaluate the expression levels of TRPV1, TRPV2, TRPV3 and TRPV4 in mucosa epithelial cells of colonic biopsies from patients with ulcerative colitis (UC) in comparison to colonic resections from non-IBD patients (control group). Immunohistochemistry, using specific antibodies and quantitative analyses of TRPV-immunostained epithelial cells, was performed in semi-serial sections of the samples. TRPV1 expression was significantly decreased whereas TRPV4 expression was significantly increased in the colonic epithelium of UC patients compared to patients in the control group (*p* < 0.05). No significant difference for TRPV2 and TRPV3 expression levels between UC and control specimens was detected (*p* > 0.05). There was no correlation between TRPV channel expression and the clinical features of the disease (*p* > 0.05). Further investigation is needed to clarify the role of TRPV channels in human bowel inflammatory response.

## 1. Introduction

The importance of the Transient Receptor Potential family (TRP) of selective and non-selective cation channels in cellular homeostasis via regulation of calcium and magnesium ions levels has been well documented [1]. TRPC (Canonical), TRPV (Vanilloid), TRPM (Melastatin), TRPA (Ankyrin), TRPN (no mechanoreceptor potential C-NOMPC), TRPP (Polycystin) and TRPML (Mucolipin) are TRP subfamilies [2]. Certain TRP channels serve as “cellular sensors” for a wide range of extracellular stimuli such as changes in temperature, osmotic pressure and pH [3]. Additionally, members of the TRP family appear to be important for the temperature-dependent formation of normal epithelial tight junctions and thus, in the control of cell proliferation and growth. Besides their well-documented role in the cell surface, TRP channels are reported to be present in intracellular membranes and are implicated in the trafficking of interactive proteins [3]. TRP activation in nerve cells enhances cell excitability leading to increased release of neurotransmitters whereas in peripheral cells (e.g., epithelial cells, immune cells), it results in increased expression of inflammatory mediators [1,2,3].

TRPV1, TRPV2, TRPV3, and TRPV4 along with TRPM8 and TRPA1 constitute the thermo-TRPion channels [4]. In particular, the highest levels of ion permeability of TRPV1 channels are achieved when they are exposed to temperatures higher than 42 °C. TRPV1 can also be activated by physical stimuli including acidic pH, mechanic distention and high membrane electric potential. Exogenous substances (e.g., capsaicin) as well as endogenous derivates like endocannabinoids (e.g., anandamide) and palmitoylethanolamide augment TRPV1 channel activity. TRPV1 expression has been found in many parts of the nervous system where it plays a crucial role in clinical conditions such as migraines, schizophrenia, myasthenia gravis, Alzheimer disease and depression [1,2,3,4,5,6]. Furthermore, TRPV1 is implicated in neurogenic inflammation, a process which involves perception of pain and both vasodilation and plasma extravasation aroused from the release of two vasoactive neuropeptides, calcitonin gene-related polypeptide (CGRP) and substance P (SP), from a subpopulation of peptidergic neurons which highly express TRPV1 [5]. TRPV2 channels share 50% domain similarity to TRPV1. They respond to noxious heat with an activation threshold of >52 °C, to changes in osmolarity and to membrane stretch. Accumulating data provide evidence that TRPV2 might participate in neurogenic inflammation [7]. TRPV3 protein produced by the translation of the same with TRPV1 gene, reaches the highest levels of its permeability when exposed to temperatures of 33–39 °C and chemical stimuli like menthol, carvacol, camphor, and eugenol. Activation of TRPV3 has been associated with cellular release of IL-1, a pro-inflammatory cytokine [8]. Temperatures of 27–34 °C, low osmolarity, acidic pH, and mechanical stress are some of the physical stimuli that increase the TRPV4 channel permeability. Certain epoxyeichosatetraenoic acid derivatives are endogenous TRPV4 agonists and phytochemical bisandrographolide A, the phorbol ester 4α-phorbol 12,13-didecanoate (4α-PDD), cannabidivarin and tetrahydrocannabivarin are exogenous TRPV4 agonists [9]. Inflammatory mediators are known to augment TRPV1 and TRPV4 activity by sensitization. Experimental data implicate TRPV1 and TRPV4 channel contribution in allodynia, thermal hyperalgesia and visceral hypersensitivity [10,11,12,13].

Ulcerative colitis (UC), Crohn’s disease (CD) and indeterminate colitis are the constituents of the inflammatory bowel disease (IBD). UC mainly affects the mucosa of the colon and rectum and is characterized by usually long-term remissions between flares and mild to severe exacerbations of abdominal pain and bloody diarrhea to weight loss, fever and anemia. During a colonoscopy, small ulcers on the colon’s lining and pseudopolyps may be revealed but the microscopic evaluation of tissue biopsies is crucial for a definite diagnosis. Increased inflammatory cells in the lamina propria, alteration of crypt architecture or even crypt abscesses and ulcers are some of the typical histopathological features of UC tissue specimens [14,15]. Despite the slightly elevated risk of colorectal cancer and the life-threatening complications of severe exacerbations, no difference in mortality rates between patients with UC and the background population has been revealed [16,17].

The impact on the quality of patients’ life with IBD on the health care system and society is of great importance and this partly explains the growing interest in involving new molecules for the treatment of the disease [18]. To that point is the investigation of TRPV1–4 channel expression in IBD patients. Previous studies have detected increased TRPV1 [19,20,21,22,23,24,25,26,27] and TRPV4 [28,29,30,31] expression in sensory fibers which was correlated with visceral hypersensitivity and hyperalgesia in inflamed human and mouse bowel. Quantitation of mRNA levels for TRPV1 [24,26,27] and TRPV4 channels [29,30] has been also assessed in colon biopsies from IBD patients and healthy controls. Recent data in experimental animals implicate TRPV2 in the development of colitis [32] whereas contribution of TRPV4 to intestinal inflammation via chemokine release has been reported [29]. The expression of TRPV1 and TRPV4 in epithelial cells of the human colon [26,29], and TRPV3 presence in distal mouse colon epithelium has been documented [33]. These findings contribute to current knowledge of nociceptive signals generated in the intestine by exciting sensitized nociceptors as a result of mechanical stimulation or distension implying that targeting TRPV channels could be a new therapeutic opportunity for treating patients with IBD [34,35,36,37].

Given the histological changes in the mucosa of patients with IBD and the involvement of TRPV channels in intestinal inflammation, we aimed to assess the immunohistochemical quantification of TRPV1, TRPV2, TRPV3, and TRPV4 channel expression in the mucosal epithelium of colonic biopsies from patients with UC compared with colonic resections from non-IBD patients (control group). The relationship between channel expression and patients’ clinical manifestations of the disease was also investigated.

## 2. Materials and Methods

### 2.1. Patients

A total of 52 Greek patients of mean age about 49.17 (±17.96) years old either treated for an exacerbation of UC or proto-diagnosed with this type of IBD (26 active, 24 quiescent and 2 with dysplasia) in the Department of Internal Medicine of “Agios Andreas” Hospital, Patras, Greece, from 1996 to 2014, were included in this study. The corresponding tissue blocks were retrieved from archival files of the Department of Pathology of “Agios Andreas” Hospital, Patras. The control group comprised of gut tissue samples from non-IBD patients (*n* = 12; mean age, 75.25 years, range, 68–83 years) excluded due to colon cancer (retrieved up to 5 cm away from the tumor’s edge), postoperative ileus, and lipomatosis of the ileocecal valve. The control tissue samples were collected from the same department, during the same period. The use of the human specimens was in accordance with the University of Patras Ethics Commission. All research protocols were conducted, and patients were treated in accordance with the tenets of the Declaration of Helsinki.

### 2.2. Immunohistochemistry

All tissues were prepared in formalin and embedded in liquid paraffin. Semi-serial sections of 4 μm collected on poly-l-lysine slides, deparaffinized in xylene and dehydrated using graded alcohol diluents up to water were used for antigen retrieval which was performed by microwaving the slides in 0.01 M citrate buffer (pH 6). Endogenous peroxidase activity was quenched by treatment with 1% hydrogen peroxide solution for 20 min. Incubation at room temperature with 1% bovine serum albumin (SERVA, Heidelberg, Germany) in Tris-HCL-buffered saline was performed for 10 min. Tissue sections were subsequently incubated with primary antibodies overnight at 4 °C for TRPV1, TRPV2, TRPV3 and 2 h RT for TRPV4. Detection of the TRPV1, TRPV2, TRPV3 and TRPV4 channels was performed using the polyclonal rabbit anti-TRPV1 antibody (cat. no. NBP1-71774; dilution 1:200; Novus Biologicals, Ltd., Cambridge, UK), polyclonal rabbit anti-TRPV2 (cat. no. TA317464; dilution 1:200, Acris Antibodies GmbH, Herford, Germany), monoclonal mouse anti-TRPV3 antibody (cat. no. AM20072PU-N; dilution 1:300, Acris Antibodies GmbH, Herford, Germany), and the rabbit polyclonal to TRPV4 (cat. no. ab39260; dilution 1:200) (Abcam, Cambridge, UK). These antibodies have been used to detect human TRPV channels in previous studies [38,39,40,41]. After three rinses in buffer, the slides were incubated with the un-avidin-biotin complex technique named Envision (Dako Cytomation; Agilent Technologies, Inc., Santa Clara, CA, USA). Tissue staining was visualized with 3,3′-diaminobenzidine (DAB) as a chromogen (which yielded brown reaction products). Slides were counterstained with Mayer’s hematoxylin solution, dehydrated and mounted. To ensure antibody specificity, negative controls included the omission of primary antibody and substitution with non-immune serum. Control slides were invariably negative for immunostaining. Renal tissue was used as positive control for TRPV1, TRPV3, and TRPV4 antibodies and ophthalmic pterygium for TRPV2 antibody [42,43].

### 2.3. Scoring

All immunohistochemical sections were assessed blindly and independently by two observers (TR and MA), followed by a joint review for resolution of any differences. The expression of proteins was determined as the mean percentage of positive mucosa epithelial cells, manually counted, with the aid of an ocular grid, in ten non-overlapping, random fields (total magnification, ×400) for each case (labeling index, LI; % labeled cells). Immunopositively stained endothelial and lamina propria cells were excluded from the cell counts. Expression of proteins included in this study was examined in adjacent (semi-serial) sections of each sample. Microphotographs were obtained using a Nikon DXM 1200C digital camera mounted on a Nikon Eclipse 80i microscope and ACT-1C software (Nikon Instruments Inc., Melville, NY, USA).

### 2.4. Statistical Analysis

Non-parametric methods were used for the statistical analysis of the results. Median comparisons were performed with Wilcoxon’s Rank-Sum test (equivalent to the Mann–Whitney U test) and the Kruskal–Wallis test. Correlation analysis was performed by utilizing Kendall’s τ (or Spearman’s ρ) rank correlation to assess the significance of associations between LIs. *p* values of <0.05 were considered to indicate a statistically significant difference. Statistical analyses were carried out using the SPSS package (version 23.0; SPSS, Inc., Chicago, IL, USA).

## 3. Results

### 3.1. Immunolocalization of Transient Receptor Potential Vanilloid Channels in Ulcerative Colitis and Control Non-IBD Samples

**Figure 1:**
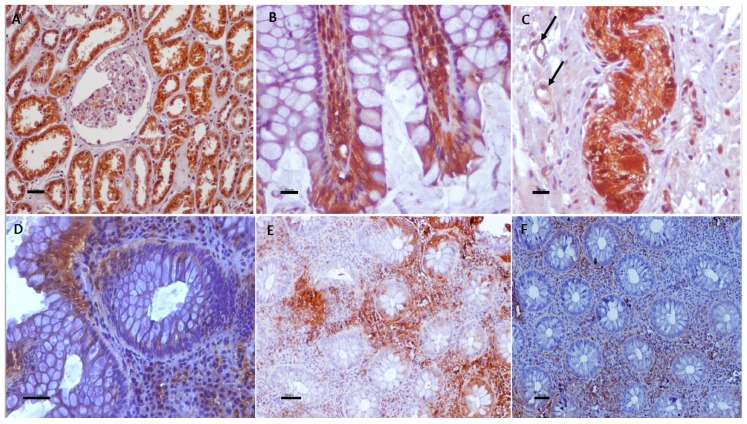
TRPV1 immunolocalization in UC and control non-IBD samples (**A**–**E**) and TRPV3 nerve fiber immunolabeling in UC (**F**). (**A**) Renal tissue sections were used as positive control for TRPV1-immunostaining; (**B**) Strong cytoplasmic TRPV1 immunoreactivity in mucosal epithelium of control group. Note TRPV1-immunostained cells in lamina propria; (**C**) Cells in enteric nervous system display strong TRPV1 immunopositivity. Furthermore, endothelial cells are TRPV1-immunoreactive (arrows); (**D**) Strong cytoplasmic TRPV1 immunostaining in a few superficial mucosa cells of this UC specimen; (**E**) Heterogeneity in TRPV1 in epithelium of UC sample. Note TRPV1-immunonegative mucosa cells nearby to TRPV1-immunopositive mucosa cells (LI = 50). (**F**) TRPV3 immunoreactivity in nerve fibers in UC. Counterstain, hematoxylin; original magnification, ×400 (**A**–**D**,**F**), ×200 (**E**); scale bar, 50 μm.

**Figure 2:**
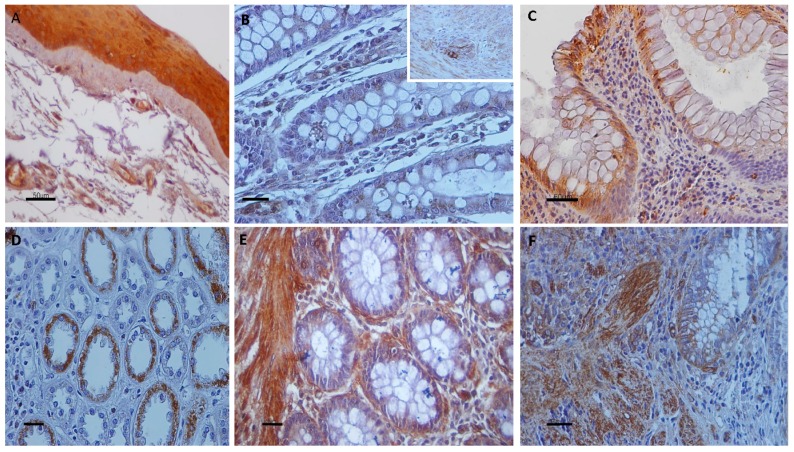
Panel presenting expression patterns of TRPV2 (**B**,**C**) and TRPV3 (**D**,**F**) in UC and control samples; (**A**) Ophthalmic pterygium tissue samples were used as positive controls for TRPV2 immunoreactivity; (**B**) TRPV2-immunostaining in intestinal epithelial cells of control colon. ((**B**), insert) Cells in enteric nervous system display strong TRPV2-immunopositivity; (**C**) Moderate cytoplasmic TRPV2 staining in numerous mucosa cells in UC specimen. Several TRPV2-immunopositive cells are observed in lamina propria cells; (**D**) Renal tissue sections were used as positive control for TRPV3-immunostaining; (**E**) Aberrant cytoplasmic TRPV3-immunostaining in epithelial cells of control sample. Note the strong-immunostained smooth muscle cells in muscularis mucosa; (**F**) Cytoplasmic expression of TRPV3 in epithelium and muscularis mucosa of UC sample. Counterstain, hematoxylin; original magnification, ×400; scale bar, 50 μm.

**Figure 3:**
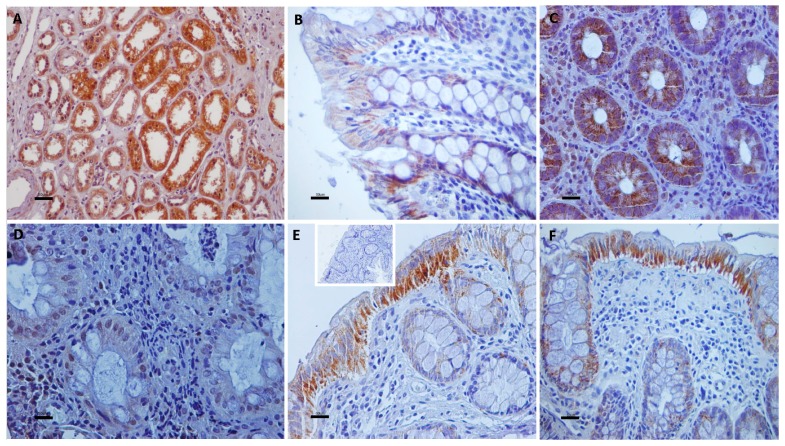
Panel depicting the cellular distribution of TRPV4 in UC and control intestine specimens. (**A**) Renal tissue sections were used as positive control for TRPV4-immunostaining. (**B**) Weak TRPV4-immunostaining in intestinal epithelial cells of control colon. (**C**) Granular cytoplasmic TRPV4-immunoexpression is identified in the cytoplasm of numerous mucosa cells in UC. (**D**) Nuclei of mucosa cells display TRPV4 immunostaining in this UC sample. (**E**,**F**) Strong granular cytoplasmic TRPV4 immunolocalization in superficial mucosa cells and goblet cells whereas there are mucosa cells with weak immunostaining of UC samples. ((**E**), insert) Immunostaining is absent in negative control sections. Counterstain, hematoxylin; original magnification, ×400; scale bar, 50 μm.

Cytoplasmic TRPV1 immunostaining was detected predominantly in the upper layer of the epithelium in 98% of UC specimens. All epithelial layers in UC cases demonstrated TRPV2, TRPV3, and TRPV4 cytoplasmic immunoreactivity, 71%, 89%, and 94% respectively. Strong cytoplasmic immunoreactivities for TRPV1, weak for TRPV2, moderate for TRPV3, and weak to moderate for TRPV4 channels were observed in epithelium of all (100%) control tissues. TRPV4 nuclear immunostaining was also noticed in certain epithelial cells. Scattered cells in the lamina propria, vascular endothelium, muscularis mucosa, and enteric nervous system displayed immunopositivity for all TRPV channels (Figure 1, Figure 2 and Figure 3).

### 3.2. Quantitative Analyses of the Immunohistochemical Findings

**Figure 4:**
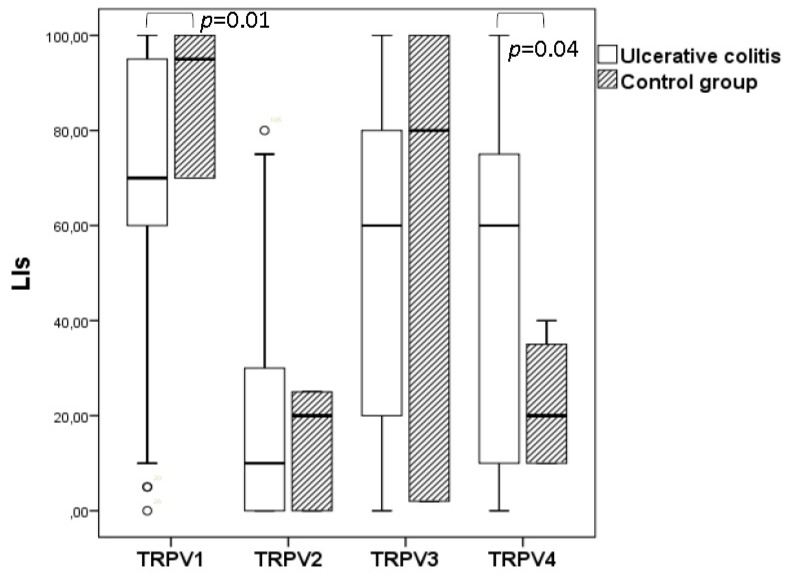
Comparison of TRPV channel expression in mucosa epithelial cells of UC and non-IBD control samples. Significant differences between UC and non-IBD group for TRPV1 and TRPV4 expression was detected (*p* < 0.05). No significant difference was observed regarding TRPV2 and TRPV3 expression (*p* > 0.05).

Immunohistochemical findings are illustrated in Table 1. TRPV1 expression levels were significantly decreased whereas TRPV4 expression levels were significantly increased in UC specimens compared with control non-IBD samples. In contrast, no significant difference was identified for TRPV2 and TRPV3 LIs between UC and control group (Figure 4). A significant correlation was found between TRPV1 and TRPV3 expression levels (Spearman’s rho = 0.462; *p =* 0.002) and between TRPV3 and TRPV4 expression levels (Spearman’s rho = 0.357; *p =* 0.01) in UC. Finally, TRPV1–4 channel expression was independent of the extent of colon inflammation, the clinical features and the symptoms of the disease as well as patients’ age and gender (*p* ≥ 0.05.)

## 4. Discussion

Previous studies have focused on quantitation of mRNA levels for TRPV channels in IBD. Thus, Kun et al. [26] demonstrated a decreased TRPV1 gene expression in UC patients and downregulation of TRPV1 transcripts in Trpa1 KO animals parallel to the enhanced inflammation upon DSS treatment. In contrast, Keszthelyi et al. [24] found no changes in mRNA levels of TRPV1 in UC patients in remission. Additionally, Fichna et al. [30] showed that TRPV4 mRNA expression was significantly elevated in patients with UC compared with healthy subjects whereas D’Aldebert et al. [29] reported no significant difference for TRPV4 mRNA quantitative expression in UC. Considering that colonic nerve fibers in IBD patients highly express TRPV1 and TRPV4 channels [19,22,28], it is obvious that these neurons largely contribute to the TRPV mRNA levels detected in colonic samples.

In the present study, TRPV channel immune-expression was quantitated in mucosal epithelium of UC and non-inflamed intestine samples (control group). The percentage of positively (labeled) cells out of the total number of epithelial cells was counted and the data was statistically analyzed. UC patients showed statistically decreased TRPV1 expression and statistically increased TRPV4 expression compared with the control group. Vinuesa et al. [44] have shown increased carcinogenesis in mice genetically deficient in TRPV1 which was strongly related to inflammation. Therefore, TRPV1 decreased expression in epithelium of UC samples may be associated with the exacerbated colon inflammation and consequently with the loss of the protective role of TRPV1 against colon cancer. However, in a similar study of Luo et al. [45] a significant upregulation of TRPV1 in colonic epithelium was observed in active IBD patients. Future investigations would clarify the involvement of epithelial TRPV1 channels in pathogenesis of IBD.

Activation of TRPV4 channels in mouse intestinal epithelial cells has been implicated in paracellular epithelial cell permeability, increased intracellular calcium concentrations and maintenance of chronic inflammation via chemokine release and recruitment of monocytes, macrophages, neutrophils and Th1 cells [29]. The increased TRPV4 expression in human mucosa epithelial cells of patients with UC may indicate a possible role of this channel in the inflammation process and provides TRPV4 as an attractive therapeutic target for human IBD. It is worthy to note that TRPV4 staining was mainly localized in the cytoplasm but there were cases in which TRPV4 immunostaining was present in the nucleus of epithelial cells. The feature of TRPV4 localization only in the nucleus has been also shown in myocardium of neonatal mice [46]. Although TRPV1 and TRPV4 were differentially expressed in inflamed bowel tissues, there was no significant correlation with clinical features of the patients and disease severity. Furthermore, the apparent difference in mean age between control and diseased groups did not influenced the data.

Low expression levels of TRPV2 were identified in both UC and normal intestine tissue and there are no published data referring to the contribution of TRPV2 in human ulcerative colitis. However, previous knowledge indicates the possible involvement of TRPV2 in experimental colitis [32]. Additionally, the role of TRPV3 in the human alimentary canal has not been well investigated. TRPV3 levels were slightly decreased in patients with ulcerative colitis in this study. It has been reported that, TRPV3 channels are important for the integrity of the epidermal barrier [8]. It would be interesting to define the contribution of TRPV3 channels in gastrointestinal inflammation and maintenance of the mucus integrity. Statistical analyses revealed the existence of positive correlation between TRPV1 and TRPV3 expression levels and between TRPV3 and TRPV4 expression levels in UC samples. Since, the heterotetramerization of TRPV1 and TRPV3 has already been documented [5], the effects of co-expression of TRPV3 and TRPV4 should be studied.

It is important to note that a variety of cells in the lamina propria exhibited TRPV immunoreactivity. Accumulating data show the presence of TRPV1 and TRPV4 in inflammatory cells including macrophages, leukocytes [26,30]. Furthermore, blood vessels demonstrated strong immunopositivity for all TRP channels. It is known that in endothelial cells, TRPV1 is activated by endocannabinoids, TRPV3 by dietary agonists, and TRPV4 by shear stress, epoxyeicosatrienoic acids and downstream of Gq-coupled receptor activation. Ca^2+^ entry through endothelial TRPV channels triggers NO^−^ and EDHF-dependent vasodilation [47,48]. Specifically, TRPV4 activation and Ca^2+^ entry may occur by mechanical stimulation of the endothelium by increased fluid viscosity and thus shear stress [49]. It would be interesting to define the role of endothelial TRPV channels in angiogenesis and carcinogenesis as recent published data implies that TRPV3, TRPV4, TRPV5, TRPM4 and TRPC6 may be thought of as potential genes contributing to colorectal cancer tumorigenesis [50]. Further investigation is needed for the TRPV channels’ involvement in IBD and any possible correlation between the expression levels of these channels and the presence of dysplasia as well as the patients’ complications and treatment, in large-scale studies.

## Figures and Tables

**Table 1:** Immunohistochemical expression of TRPV1, TRPV2, TRPV3, and TRPV4 channels in colonic epithelium of human UC and non-IBD control samples. The (non-parametric) Wilcoxon’s Rank-Sum test was performed and the level of significant was defined as *p* < 0.05.

TRPV LIs	Ulcerative Colitis (*n* = 52)	Control Group (*n* = 12)
**TRPV1 LIs** Mean ± SD, % (range)	68.333 ± 28.28 ^a,b,c^ (0–100)	88.33 ± 16.07 ^f,g,h^ (70–100)
**TRPV2 LIs** Mean ± SD, % (range)	18.52 ± 23.77 ^d,e^ (0–80)	15.00 ± 13.22 ^i^ (0–25)
**TRPV3 LIs** Mean ± SD, % (range)	51.00 ± 36.19 (0–100)	60.66 ± 51.78 ^k^ (2–100)
**TRPV4 LIs** Mean ± SD, % (range)	47.80 ± 33.09 (0–100)	22.50 ± 15.00 (10–40)

LI, the percentage of positive-labeled cells from the total number of epithelial cells counted; Mean, mean labeling index; SD, standard deviation; ^a^ *p* < 0.001 vs. TRPV2 expression in UC; ^b^ *p* = 0.01 vs. TRPV3 expression in UC; ^c^ *p* = 0.002 vs. TRPV4 expression in UC; ^d^ *p* < 0.001 vs. TRPV3 expression in UC; ^e^ *p* < 0.001 vs. TRPV4 expression in UC; ^f^ *p* < 0.001 vs. TRPV2 expression in control group; ^g^ *p* < 0.02 vs. TRPV3 expression in control group; ^h^ *p* < 0.001 vs. TRPV4 expression in control group; ^i^ *p* < 0.001 vs. TRPV3 expression in control group; ^k^ *p* = 0.01 vs. TRPV4 expression in control group.
